# Tag-free indoor fall detection using transformer network encoder and data fusion

**DOI:** 10.1038/s41598-024-67439-2

**Published:** 2024-07-21

**Authors:** Muhammad Zakir Khan, Muhammad Usman, Jawad Ahmad, Muhammad Mahboob Ur Rahman, Hasan Abbas, Muhammad Imran, Qammer H. Abbasi

**Affiliations:** 1 University of Glasgow, James Watt School of Engineering, Glasgow, G12 8QQ UK; 2 Artificial Intelligence Research Centre, Ajman University, Ajman, UAE; 3 School of Computing, Engineering and Built Environment, Glasgow Caledonian University, Glasgow, G4 0BA UK; 4 Cybersecurity Center, Prince Mohammad Bin Fahd University, Al-Khobar, 31962 Saudi Arabia; 5 Electrical engineering department, Information Technology University, Lahore, Pakistan

**Keywords:** Health care, Energy science and technology, Engineering

## Abstract

This work presents a radio frequency identification (RFID)-based technique to detect falls in the elderly. The proposed RFID-based approach offers a practical and efficient alternative to wearables, which can be uncomfortable to wear and may negatively impact user experience. The system utilises strategically positioned passive ultra-high frequency (UHF) tag array, enabling unobtrusive monitoring of elderly individuals. This contactless solution queries battery-less tag and processes the received signal strength indicator (RSSI) and phase data. Leveraging the powerful data-fitting capabilities of a transformer model to take raw RSSI and phase data as input with minimal preprocessing, combined with data fusion, it significantly improves activity recognition and fall detection accuracy, achieving an average rate exceeding $96.5\%$. This performance surpasses existing methods such as convolutional neural network (CNN), recurrent neural network (RNN), and long short-term memory (LSTM), demonstrating its reliability and potential for practical implementation. Additionally, the system maintains good accuracy beyond a 3-m range using minimal battery-less UHF tags and a single antenna, enhancing its practicality and cost-effectiveness.

## Introduction

Falls are the second most prevalent cause of accidental injury world wide, following traffic accidents^1^. For individuals aged over 65, falls are the leading cause of injuries, contributing significantly to mortality, morbidity, frailty, and disability^2^. Annually, approximately 30% of the elderly experience at least one fall, with 90% of these incidents leading to hip fractures and 60%3 resulting in head injuries^1^. Moreover, about half of the elderly who fall endure prolonged periods on the ground, known as long-lie, which increase their risk of severe complications such as pressure sores, hypothermia, pneumonia, dehydration, and potential fatality^3^. Currently, fall detection methods can be broadly categorised into two main approaches: wearable^4–7^ and non-wearable^8–10^. Wearable-based fall detection relies on sensors like gyroscopes and accelerometers attached to the body to monitor changes in activity. In contrast, non-wearable-based approaches include visual fall detection^8,11^, environmental fall detection, and radio frequency identification (RFID) based fall detection^12^. Visual detection employs cameras to identify falls and monitor activities such as running and walking. Environmental fall detection uses strategically  placed sensors, like predictors and infrared sensors, within the user’s activity area to effectively detect falls. In contrast, RFID-based fall detection utilises RFID tags to detect falls and has gained attention due to its affordability, lightweight design, small form factor, and battery-less operation. Previous studies have primarily focused on tag-based approaches^13–16^ where RFID tags were attached to humans for activity capture. This includes, object identification and tracking^17–20^, which can infer human activities from RFID-labeled objects^21,22^. However, traditional tag-based systems require specific objects or users to have RFID tags attached to track their movement and infer various activities. This becomes a limitation when activities such as pushups, walking, and body rotation do not involve interaction with RFID-tagged products or cause environment movement.

Recently, tag-free solutions have emerged as a promising alternative to address the inconvenience and intrusiveness of attaching RFID tags directly to humans^23–25^. Instead of attaching tags, multiple stationary RFID tags are strategically deployed as reference points, with their readings influenced by nearby human activities. This innovative approach enables the identification of various activities by analysing the backscattered signals from these reference tags. However, surrounding human activity, such as obstructing the line of sight (LOS) path, can introduce interference in RFID signals, despite the inherent reliability of the devices. To address this issue, researchers have turned to deep learning approaches for automatic analysis of signal fluctuations^25–27^. These methods aim to extract meaningful features from RFID signals, requiring substantial amounts of training data to construct robust deep neural networks. Nonetheless, these approaches are sensitive to environmental changes^26,28^. For instance, the TagFree system^27^ uses neural networks to analyse data derived from measuring the angle of arrival (AoA) of multipath signals using specialized antenna arrays. However, in the presence of environmental changes, these neural networks require periodic updates and retraining with new data, resulting in significant data collection and training overhead during practical implementation. In addition to detecting falls, identifying pre-fall features such as dizziness or staggering can improve overall prediction accuracy and provide early warnings. These behaviours are necessary for fall prevention and timely intervention.

Introducing our tag-free fall detection ‘*TFree-FD*’ system that leverages a passive RFID tag array and a neural network for precise and reliable fall detection. We propose an RFID-based method using a transformer architecture that offers a privacy-preserving, cost-effective, and robust solution for indoor fall detection. Unlike conventional approaches that rely solely on raw data, our system captures raw radian and RSSI information from multiple tags. This data is preprocessed into spectrum frames, from which key features are extracted to classify activities accurately, including normal falling, walk falling, standing, and leaning. Our *encoder-only* transformer model employs the attention mechanism^29^, significantly reducing training time compared to recurrent neural architectures such as long short-term memory (LSTM) and convolutional neural networks (CNN)^30^. The fusion of RSSI and phase information generates characteristic data essential for fall detection. A key advantage of our approach is its compatibility with existing RFID readers for indoor activity detection. This makes it a cost-effective and practical solution, implementable with off-the-shelf RFID readers, requiring only a single ultra-high frequency (UHF) reader with a single circular polarised antenna. Our approach uniquely integrates both RSSI and phase data using a transformer-based deep learning model, distinguishing it from existing methods that typically rely on a single type of data.

## Related work

In recent years, RFID technology has gained prominence as an affordable, compact, non-line-of-sight (NLoS) sensing solution with easy deployment, battery-less operation, and low maintenance requirements. This widespread adoption spans various applications, with particular emphasis on fall detection within the elderly population. Research in this area has explored diverse technologies, including vision-based, wearable, and wireless signal-based approaches. Vision-based fall detection systems evaluate red, green and blue (RGB) or depth images captured from single cameras, camera arrays, or depth camera systems. Despite advancements in both RGB and depth systems, challenges persist in distinguishing falls from activities resembling falls, such as lying down or leaning. Addressing the identification of falling posture, several studies have proposed distinct approaches. For example, Rougier et al.^31^ examined how the human body’s contours deform in captured images to detect falls. Mirmahboub et al.^32^ employed cameras to monitor falls in elderly, extracting behavioural variables from video sequences for fall identification amidst normal daily activities. The study conducted by^33^ constructed a 4D-CNN network that processes multiple modal data individually in respective CNN streams for fall detection. Another study by^34^ used RetinaNet to identify and track moving subjects in video frames and employed characteristics extracted by enhanced MobileNets to categorise human motion as ‘falling’ or ‘not falling’. These approaches offer benefits such as not requiring the wearer to carry a device, providing intuitive vision, and detecting falls from various perspectives. However, they demand high-performance devices due to strict requirements for algorithm efficiency in image processing. Moreover, they might raise concerns about user privacy and are sensitive to changes in light intensity. Implementing these methods in complex environments is also challenging as they require an unobstructed view between the user and the camera. Despite needing unobstructed signal paths, RFID technology offers several advantages over cameras. These advantages include better privacy (no picture or video capture), lower cost and easier deployment, signal penetration through non-metallic surfaces, and consistent performance in all lighting conditions^35^. These factors make RFID suitable for indoor fall detection.

Wearable devices offer a comprehensive monitoring system capable of collecting diverse data types to depict basic daily activities. This includes the integration of wearable sensors like accelerometers, gyroscopes, and RFID technology. For instance, Le and Pan^36^ developed a fall detection system for the elderly utilising integrated wearable acceleration sensors, addressing to their specific needs. Similarly, Vallejo et al.^37^ employed a multilayer perceptron for binary classification of data from three-coordinate accelerometers. Micucci et al.^38^ categorised data using K-nearest neighbour (KNN) and support vector machines (SVM) with prior datasets. Another approach involved attaching two wearable accelerometers to elastic sportswear on the right thigh and belly^39^, utilising Bluetooth to transmit sensor data to a laptop for activity identification and fall detection. To distinguish between normal and fallen states of the human body, Wang et al.^40^ designed a pendant necklace containing a three-axis accelerometer coupled with a barometric sensor detector. Xiaoling et al.^41^ utilised the acceleration and gravity sensors of a cell phone to recognize human gestures by leveraging temporal aspects in the data. While these techniques boast impressive identification accuracy, they require users to wear sensors and other equipment at specific body locations.

Radio frequency (RF) based systems, including WiFi, radar, and RFID, offer potential solutions to address the limitations of wearable devices and cameras. Among these, WiFi systems have shown promise in fall detection, leveraging channel state information (CSI). For instance, Wang et al.^42^ demonstrated real-time automatic data segmentation and fall detection using fine-grained CSI data from WiFi devices. Another notable system, *WiFall*^43^, utilised WiFi-CSI for fall detection by measuring predefined motions and employing a one-class SVM and RF for classification. While WiFi signal detection effectively addresses challenges related to lighting and user privacy, its deployment costs can be prohibitive due to sensitivity and stability issues in complex monitoring environments, thus limiting its widespread implementation for the elderly^44^. On the other hand, Doppler radars have also been employed for fall detection based on human movement speed. However, they can be influenced by non-fall activities. To address this, Ma et al.^45^ proposed the use of ultra-wideband (UWB) monostatic radar and an LSTM algorithm for fall detection. Nevertheless, the system’s adaptability to new individuals and environments is constrained by residual environmental impacts. Tian et al.^46^ presented a solution involving two perpendicular angle-range heat maps to differentiate between human daily activities and falls, leveraging a large-scale dataset under various scenarios to mitigate environmental impact. However, the hardware and dataset requirements render this system costly and impractical in certain scenarios.

Research has explored monitoring elderly patients using battery-less RFID tags. One approach involved embedding RFID tags into clothing and placing an RFID reader on the body, using the fluctuation of RSSI values to identify different activities^47^. Toda et al.^48^ deployed RFID tags on wearable shoes and used machine learning (ML) algorithms for fall identification, but their system lacked sudden fall detection capability. The *TagFall*^49^ and *Wear-free*^50^ methods proposed to distinguished between falls and everyday activities based on sudden changes in RSSI values. Similarly, Takatou^51^ utilised passive RFID sensor tags to detect falls by analyzing pressure values and RSSI, employing random forest for real-time activity recognition, including walking and falling on stairs. Chen et al.^52^ developed an intelligent fall detection system for hemodialysis patients by enhancing the 2NN-RFE approach based on a residual feature extraction algorithm. The mobility and fall patterns of the elderly were also identified by Zhu et al.^53^ using commercial RFID readers and wavelet transform and ML algorithms. Despite leveraging ML and DL approaches, these methods have drawbacks. Challenges include difficulty in identifying isolated incidents, system detection delays, and the need for expertise. The ML methods have limited capacity to extract inclusive features from outcomes. Additionally, the convolution operation is constrained by the receptive field, LSTM models are prone to overfitting, and training adversarial networks poses further challenges.

In this study, we leverage an attention-based transformer network^29^, which entirely avoids the use of a decoder, recurrence, and convolutions. The transformer-based models were further studied for human activity recognition (HAR) using wearable and contactless sensors. For instance, recent study has demonstrated the effectiveness of lightweight transformers in processing data from wearable sensors for HAR and achieved improved accuracy and efficiency using smart phone^54^. Another study used transformers in wearable devices for healthcare applications^55^. These studies demonstrate the flexibility of transformers for various applications, supporting our work’s originality in fall detection using RFID in a contactless manner. Furthermore, TransTM^56^, a contactless method for detecting activities, uses four antennas to collect RSSI information and processes it within a 3-meter distance. However, it excludes the significance of phase data, which can enhance activity recognition accuracy. Our proposed *TFree-FD* method fuses RSSI and phase data using a single antenna and minimum number of RFID tags, extending the detection range to 3.5–4.5 m in noisy environments. While TransTM adopts an encoder-decoder structure, which makes the model complex, we simplify it by using an encoder-only structure, reducing complexity and boosting efficiency for contactless daily activities and fall detection. The proposed method uses multi-head self-attention with residual connections to create feature representations. We chose the transformer architecture because it has been proven to be accurate in tests, especially when dealing with global patterns in signals. Using a transformer helps us get important information from the signals, which makes our RFID-based fall detection system better. The model’s performance is shown to be excellent in our experiments. Notably, the model gets a remarkable 96.5% average accuracy on fused RFID data, which is better than other methods.

## Performance evaluation

This section’s aim is to assess the fall detection system’s performance by employing diverse features, including RSSI, phase, and fusion of RSSI and phase. The effectiveness and accuracy of the proposed contactless RFID-based fall detection approach, utilising an early fusion transformer model, will be evaluated through a set of comparative experiments.

### Activity recognition methods

Activity recognition methods play an important role in precisely detecting and classifying human daily living activities, especially in indoor environments. In this subsection, we explore three essential approaches for activity recognition: *Activity Recognition using RSSI*, *Activity Recognition using Phase*, and *Activity Recognition using Fusion*. These approaches greatly enhance the accuracy and comprehension of various human activities within an indoor environment. In our study, we have categorised activities into five distinct classes in order to include the range of movements relevant to daily activity and fall detection. The explanation of these activities is as follows:

1. **No Activity:** No human subject in the activity area.
2. **Standing: ** The action of ‘Standing’ from Sitting (static) position in the activity area.
3. **Sitting: ** The action of ‘Sitting’ from Standing (static) position in the activity area.
4. **Leaning: ** Leaning forward with the upper body in the activity area.
5. **Normal Fall:** The action of a subject falling to the ground from a standing position.
6. **Walk Fall:** The action of subject stumbling or slipping while walking or in motion.

#### Activity recognition using RSSI

The literature extensively discusses the use of passive UHF RFID tags for indoor activity recognition, particularly in fall detection. These RFID tags are activated by readers using air interface protocols (EPC Class 1 Gen-2 and ISO-18000-6c) for data transmission and reception^57^. During practical scenarios, passive RFID tags provide raw data to the reader in a 5-tuple format, consisting of RSSI, timestamp, EPC, TID, and frequency. To generate an RSSI dataset, several preprocessing steps are performed as outlined in Sect. 5.2.

**Algorithm 1:**
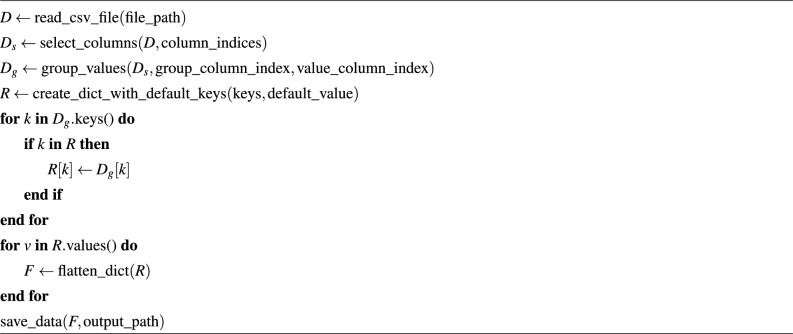
Pseudo code for RSSI data saving and grouping

In this study, the recognition of falls’ activities is conducted using a carefully designed experimental setup as depicted in Fig. 8. A designated area in front of the *TRG-Wall* was used to perform five distinct activities, and the data collected for each activity is presented in a tabular format. To provide a comprehensive analysis of the collected data, we employed both column-wise and row-wise presentations. Column-wise visualization proved effective in illustrating standing and leaning activities, while the row-wise representation is better suited for depicting falls and fall-related activities. Specifically, this was observed in relation to normal falls and walking falls, with a particular emphasis on rows 2nd and 3rd. In contrast, columns 1st and 2nd primarily depicted leaning and standing activities, exerting minimal influence on the remaining columns.

**Figure 1:**
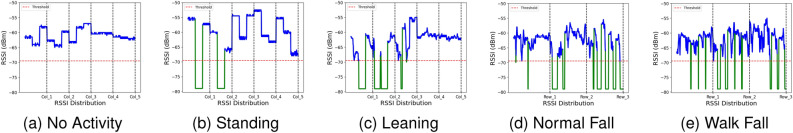
Analysis of falling activity recognition: distribution and magnitude of RSSI (row-wise and column-wise).

Building upon these insights, the compelling evidence supporting accurate fall and fall-related detection in shared spatial environments is presented in Fig. 1. Recorded RSSI strengths ranged from $-50$ to $-69$ dBm, with obstructed tags leading to decreased RSSI readings. The red dotted line serves as an activity recognition threshold, set to address scenarios involving non-reading or tag-blocking. The use of green highlights instances of RSSI data reading obstruction or activity recognition challenges. Specifically, Fig. 1b,c depict RSSI values for standing and leaning activities, respectively, in rows $2^{nd}$ and $3^{rd}$. Meanwhile, Fig. 1d,e showcase normal and walking fall activities performed in the same location.

**Figure 2:**
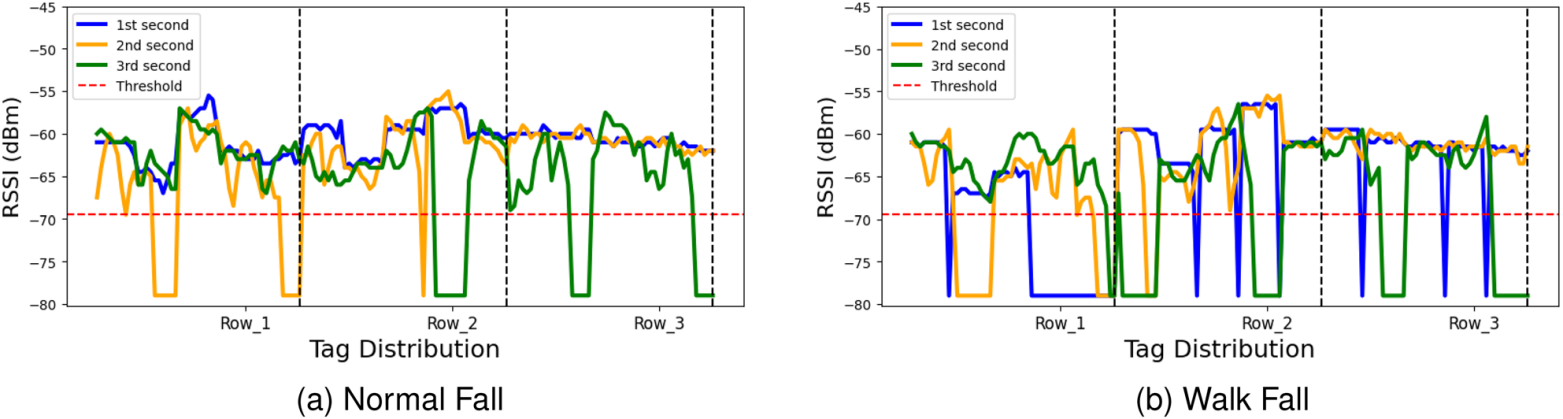
Visual representation: analysis of falling per second.

To enhance the representation of falling events, we split the RSSI data linked with three-second falling patterns into one-second intervals and employ distinct color codes for visual clarity. The color scheme is as follows: blue for the 1st second, orange for the 2nd second, and green for the 3rd second of the falling pattern data. This visual representation effectively demonstrates the sequential progression of the fall activity. For instance, in Fig. 2, the initiation of the fall by the subject is evident during the 1st second (blue color) from row-1 to row-3. This is followed by movement observed during the 2nd second (orange color) and the 3rd second (green color) from row-2 to row-3, signifying the progression of the fall activity in a row-wise manner.

#### Activity recognition using phase

RF backscatter technology enables bidirectional signal transmission over a distance of 2*d*, making it possible to monitor human activity by analyzing phase differences in RF features using cross-correlation. The relationship between distance, antenna phase rotation ($\theta _{\text {Ant}}$), and tag phase rotation ($\theta _{\text {Tag}}$) can be mathematically described as a periodic function with a phase shift of ($2\pi$) radians occurring every $\lambda /2$ in the RF communication distance. Accurate evaluation of phase difference calculations during activity is essential for assessing their discriminative nature.

1 $$\begin{aligned} \theta = \left( \theta _{\text {Ant}} + \theta _{\text {Tag}} + 2\pi \frac{2d}{\lambda }\right) \bmod (2\pi \lambda ). \end{aligned}$$

The correlation coefficient $r_{xy}$ quantifies the association between two activities, each represented by 36 phase readings. It is calculated as:

$$\begin{aligned} r_{xy} = \frac{\sum (x_i - {\bar{x}})(y_i - {\bar{y}})}{\sqrt{\sum (x_i - {\bar{x}})^2 \sum (y_i - {\bar{y}})^2}}. \end{aligned}$$

Here, $x_i$ and $y_i$ are phase readings, and ${\bar{x}}$ and ${\bar{y}}$ are their respective means. The numerator sums the products of deviations from the means, and the denominator involves the square root of the products of the sums of squared deviations. This computation measures the extent of association between the activities, indicating their correlation.

**Figure 3:**
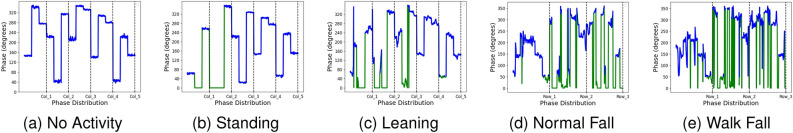
Phase-based analysis of falling activity recognition: row-wise vs column-wise.

**Figure 4:**
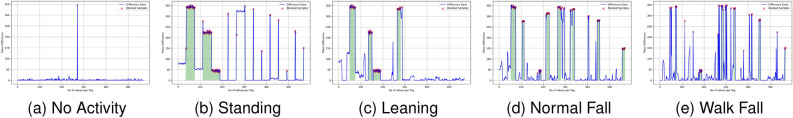
Phase difference-based analysis of falling activity recognition: row-wise vs column-wise.

Figure 3 showcases the analysis of falling activity recognition, both row-wise and column-wise, utilising phase information. Figure 4 illustrates the significance of phase difference patterns in fall activities. These patterns were obtained using the *numpy* function *np*.*corrcoef*(*x*, *y*), which quantifies the cross-correlation between two sets of data. For instance, in Fig. 4b, the standing activity demonstrates the phase difference between an empty state and a standing position. The red cross indicates the detection or blockage of phase, signifying the occurrence of activity detection. The resulting cross-correlation difference pattern proves to be an effective method for modeling activities. Notably, the smooth variation of phase differences observed when blocking tags were present during sitting activities emphasizes the accuracy and reliability of the measurements.

#### Activity recognition using fusion

Activity recognition through fusion involves integrating data from multiple modalities to enhance accuracy, specifically by leveraging both RSSI and phase-based information to optimize activity recognition algorithms. This fusion process can be accomplished using two primary concepts: early fusion and late fusion.

**I. Early Fusion in RFID-based Activity Recognition:** Early fusion, or feature-level fusion, combines unprocessed RSSI and phase data from RFID tags before classification. This integration reduces redundancy and complexity in features, resulting in more efficient models for quicker inference and reduced computational overhead. It enhances accuracy, robustness, and adaptability in RFID-based human activity detection, enabling effective recognition of various activities in indoor environments.

The algorithm for early fusion in RFID-based activity recognition involves the following steps:

1. Initialize empty datasets for RSSI and phase data: ${\textbf{D}}_{\text {RSSI}}$ and ${\textbf{D}}_{\text {Phase}}$.
2. Preprocess RSSI and phase data separately to create ${\textbf{D}}_{\text {RSSI}}$ and ${\textbf{D}}_{\text {Phase}}$.
3. Combine ${\textbf{D}}_{\text {RSSI}}$ and ${\textbf{D}}_{\text {Phase}}$ to create ${\textbf{D}}_{\text {Combined}}$ containing integrated information.
4. Extract features from ${\textbf{D}}_{\text {Combined}}$ using the *feature_extraction* method, resulting in ${\textbf{F}}_{\text {Combined}}$.
5. Perform classification on ${\textbf{F}}_{\text {Combined}}$ to predict activity labels, storing them in ${\textbf{Y}}_{\text {Pred}}$.

The algorithm for early fusion in RFID-based activity recognition can be concisely summarized as follows:

**Algorithm 2:**
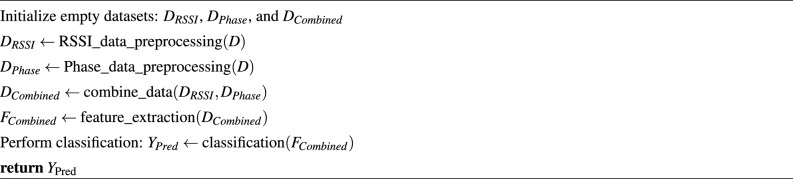
Pseudo code for early fusion

**II. Late Fusion with merit in RFID-based Activity Recognition:** Late fusion, or decision-level fusion combines independent decisions from RSSI and phase data for falling and daily living activity prediction. Separate classifiers are trained for each modality (RSSI and phase). One classifier recognizes falling patterns using RSSI, and the other relies on phase data. These classifiers make independent predictions. Late fusion employs two methods: voting-based and merit-based. In the voting-based approach, the final decision is based on majority votes from individual classifiers while merit-based fusion optimizes modality weights based on performance and reliability measures. We use merit-based to avoid bias in classifiers. This approach leverages the complementary information from RSSI and phase data, enhancing robust falling event detection, especially in complex indoor environments. The pseudo-code of late fusion with a merit-based algorithm is summarized as follows:

**Algorithm 3:**
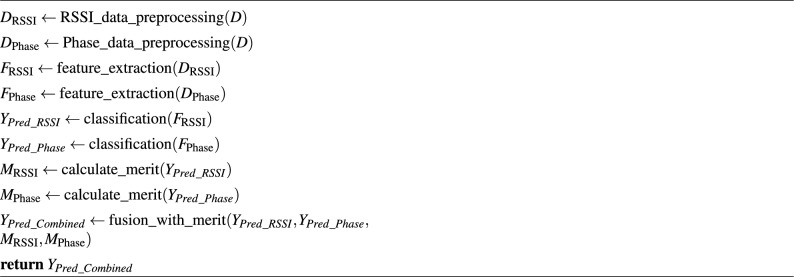
Pseudo code of merit-based late fusion

### Experimental results

We evaluated our contactless *TFree-FD* method using an 80 : 20 train-test split (random state: 42), with 32 batch size, 50 epochs, and a dropout rate of 0.01 during training. For a comprehensive view of network hyperparameters, disussed in Table 1. Our approach was compared against four prominent deep learning models: RF-finger^58^, LiteHAR^59^, Tagfree^27^, and Dense-LSTM^60^. These models represent benchmarks for device-free RF-based activity detection using traditional deep-learning methods

**Table 1:** Proposed transformer model hyperparameters.

S. no	Components	Parameters
1	Layer normalization	gamma (1) and beta (1)
2	Multi-head attention	Kernel $(1 \times 4 \times 64 )$, bias $(4 \times 64)$
3	Dense-gelu	Kernel $(100 \times 32)$, bias (32)
4	Dense-softmax	Kernel $(32 \times 10)$, bias (32)
5	Conv1D	Kernel $(1 \times 1 \times 64 )$, bias $(4 \times 64)$, ReLU

#### Overall performance of TFree-FD

**Figure 5:**
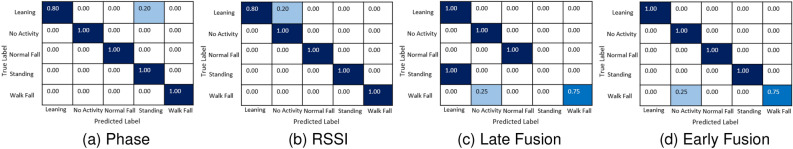
Visualization of the normalised confusion matrix pre and post fusion.

The effectiveness of the *TFree-FD* technique, which employs a transformer, is demonstrated in Fig. 5. The confusion matrix in the figure reveals a distinct diagonal pattern for RSSI, phase, early fusion, and late fusion results. In Fig. 5a achieves an accuracy of $93.3\%$. Similarly, Fig. 5b achieves $94\%$ accuracy for RSSI, while the transformer model fused with late fusion in Fig. 5c shows $86.2\%$. Remarkably, the late fusion in Fig. 5d achieves the highest accuracy at $96.5\%$. Comparing these results to the baselines in Table 2, the transformer outperforms significantly, consistently achieving the highest accuracy and F1 score across all categories. Specifically, it establishes new benchmarks on our experimental dataset, surpassing RF-finger, Dense-LSTM, LiteHAR, TagFall, CFD, and Tagfree by $11.1\%$, $6.5\%$, $5.5\%$, $4.8\%$, $4.8\%$, $1.6\%$, $4.5\%$, and $3.7\%$ in average accuracy, respectively. Moreover, our proposed F1-score outperforms RF-finger, TagFall, Tagfree, Dense-LSTM, and LiteHAR by 12.1, 8.7, 5.6, 4.0, 4.0, 1.8, 3.7 and 2.9, respectively. These results underscore the exceptional performance of our proposed encoder-based transformer architecture in fall detection.

**Table 2:** Comparing performance of proposed model with baselines.

Year	Appraoch	Activities	Accuracy (%)	F1 score
2018	RF-finger^58^	Gesture (rotate left and right, zoom in and out, swipe left and right)	85.4	83.6
2020	Dense-LSTM^60^	Walk, run, lie down, jump, and play piano	90	87.0
2022	LiteHAR^59^	Lie down, fall, walk, run, sit down, and stand up	91	90.1
2015	TagFall^49^	Fall left, right, back and forward, sit, stand, lying and walking	91.7	91.7
2024	TagFall^61^	Sitting, jumping, walking, crouching and sudden fall	91.7	91.7
2023	CFD^62^	No-activity, standing and fall	95	0.9
2018	FD-NPP^63^	Walk, stand, fall left and right	92	92
2018	Tagfree^27^	Sit, stand, bow, wave, walk, work and run	92.8	92.8
2024	TFree-FD	No-activity, standing, sitting, normal fall and walk fall	**96.5**	**95.7**

Significant values are given in bold.

#### Comparative analysis of existing methods

We compared our fusion-based transformer model with common models (CNN, RNN, LSTM) using accuracy and prediction time. Results in Tables 3 and 4 show our model’s superior accuracy and F1-scores (epochs = 50). Unlike CNN, RNN, LSTM using convolutional layers, our transformer model stands out with self-attention mechanisms for better accuracy. However, due to its extensive self-attention use and parameters, the transformer requires more computational resources, leading to less efficient prediction times compared to other methods.

**Table 3:** Comparing accuracies among different algorithms.

Algorithms	Scenario-1 moderate SNR (3.5)		Scenario-2 high SNR (4.5)	
	RSSI (%)	Phase (%)	RSSI (%)	Phase (%)
CNN	82.3	79.5	73.7	71.9
RNN	85.1	78.2	77.5	70.7
LSTM	88.8	75.1	80.6	74.8
Transformers	94	93.3	84.1	77
Transformers (late fusion)	86.2		81.4	
Transformers (early fusion)	**96.5**		**87.9**	

Significant values are given in bold.

**Table 4:** Comparing F1-scores among different algorithms.

Algorithms	Scenario-1 moderate SNR (3.5)		Scenario-2 high SNR (4.5)	
	RSSI	Phase	RSSI	Phase
CNN	0.64	0.58	0.46	0.43
RNN	0.70	0.56	0.54	0.41
LSTM	0.78	0.50	0.60	0.48
Transformers	0.88	0.86	0.68	0.54
Transformers (late fusion)	0.72		0.62	
Transformers (early fusion)	**0.93**		**0.74**	

Significant values are given in bold.

#### Comparing head count’s influence on transformer model accuracy

The effectiveness of transformer models is notably impacted by the number of attention heads within their architecture. This effect is demonstrated in Table 5, offering a comparative accuracy analysis for different head counts (epoch = 10). The accuracy of the model fluctuates with varying head counts, highlighting the influential role of headcount in determining model effectiveness. The results reveal a discernable pattern where increasing headcount generally improves accuracy, although this relationship may not be strictly linear. For example, as headcount increases from 2 to 6, accuracy consistently rises, peaking at $100\%$. However, further increasing the headcount to 8 leads to a slight accuracy reduction. This underscores the complex interplay between model complexity, attention mechanisms, and accuracy. Therefore, consideration of headcount is important when optimizing transformer models for optimal performance.

**Table 5:** Comparing number of heads (epoch =10).

Number of heads	2	4	6	8	10
Accuracy	76.3%	92.5%	100%	86.0%	81.2%

#### Comparing convolution utilization and analyzing 1D and 2D techniques’ impact on accuracy

We conducted an extensive study to compare the impact of utilizing 1D and 2D convolutions on model accuracy. The analysis spanned ten epochs and employed four attention heads. The results, presented in Tables 6 and 7, reveal a significant accuracy difference between the two convolution types across epochs. Specifically, 1D convolution demonstrated robust accuracy at 92.5%, surpassing the comparatively modest 68.1% accuracy achieved by 2D convolution. This contrast underscores the intricate interplay of convolution methodologies and underscores the pivotal role of selecting an appropriate technique to enhance transformer model precision.

**Table 6:** Comparing 1D and 2D convolution (epochs = 10 vs 50 and heads = 4).

Convolution configuration	Epochs = 10		Epochs = 50	
Convolution type	1D	2D	1D	2D
Accuracy	92.5%	68.1%	96.5%	75.4%

**Table 7:** Comparing epochs vs accuracy’s.

Epochs	10	30	40	50	70	100
Accuracy	91.9%	90.7%	93.7%	96.5%	100%	100%

## Discussion

This study thoroughly examined four deep learning models: CNN, RNN, LSTM, and the attention-based transformer. It combined early and late fusion techniques to recognise daily activities including falls using RFID data in two different scenarios. The study included three specific approaches (RSSI, Phase, and fusion) described in Sect. “Activity recognition methods”. Results showed that the transformer model performed best with the early fusion technique, outperforming CNN, RNN, and LSTM in both scenarios. The scenarios involved placing a reader and antenna 3.5 m from the subject, with an additional subject-to-wall distance of 0.5 m. A detailed analysis of the model’s performance shows that it can accurately identify most activities with over 99% accuracy. However, it struggled to distinguish between ‘walk fall’ and ‘leaning’ activities. This suggests that the model could be useful for practical applications, as shown in Table 3. It’s important to note that the model’s effectiveness, especially when combined with early fusion for contactless RFID human activity recognition, depends on the distance between the TRG-Wall and the reader antenna. We understand that the current study may not accurately represent the performance of the proposed approach in diverse real-world scenarios, even while it shows its effectiveness in a controlled single-environment setup. In order to validate the model’s robustness across various environments, future study will focus on comprehensive multi-environment testing. Furthermore, we tested our system in a realistic setting with obstacles to mimic real-world scenarios. The system performed well, accurately detecting falls even in the presence of these obstacles. This proves that our system can adapt to complex and obstacle-filled environments, making it reliable for practical use.

## Data and methods

**Figure 6:**
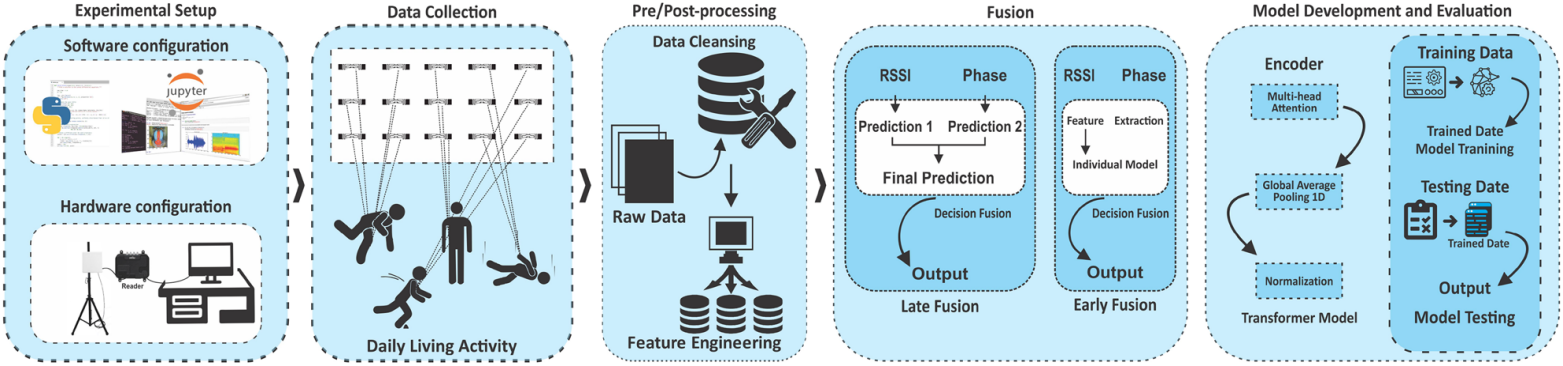
Integrated workflow for intelligent experimentation: from experimental setup to transformer model development.

This section presents an extensive overview of the methodologies and materials utilised in the experimental setup for data collection, specifically aimed at predictive analytics using deep learning techniques. Before applying these techniques, two test scenarios were designed to facilitate the data collection process. The below subsections provide detailed information on the hardware and software components meticulously organised and employed to capture RSSI and phase information from the RFID UHF passive tags array. Our proposed methodology, comprising five major components, is illustrated in Fig. 6, with each component thoroughly explained in the subsequent sections.

### Experimental setup and procedure

In designing our study, we ensured the methods and setup were relevant to the elderly population. The activities we selected (standing, leaning, falling) reflect common movements and fall scenarios among elderly individuals. To ensure safety and ethical considerations, we used volunteers to mimic these activities accurately, rather than involving actual elderly participants. We designed our system to be responsive to the types of falls that are most prevalent in the elderly population. Our system utilises RFID tags that do not require the elderly to wear any devices, improving their comfort and willingness to participate.

The experiments in this study were conducted within a dedicated $10 \times 10$ m$^2$ room in the James Watts South building at the University of Glasgow. The study received ethical approval from the University of Glasgow’s Research Ethics Committee (approval nos.: 300200232, 300190109), and all methods were performed in accordance with the relevant guidelines and regulations provided by the committee. All subjects provided written, informed consent prior to data collection. The setup comprised a $1.5 \times 1.5$ m$^2$ Transparent RFID Grid Wall (*TRG-Wall*) and a commercial UHF RFID reader. This *TRG-Wall* was strategically equipped with metal storage boxes to create rich multi-path features and ensure a robust NLoS environment. The inclusion of these obstacles was intentional to simulate a realistic, complex indoor environment, thereby inherently testing the model’s ability to maintain accuracy in the presence of obstructions. The *TRG-Wall* was divided into five columns and three rows, resulting in a total of fifteen tags being deployed. A circularly polarised antenna was positioned at horizontal distances of 3.5 and 4.5 m from the center of the *TRG-Wall*, maintaining a fixed distance of 0.5 m from the subject. The antenna height remained consistently set at 0.75 m above the floor surface. The subject was instructed to perform various activities at designated locations, while data collection was focused on both the subject’s activities and the surrounding environment characteristics. The data collection setup was comprised of two main components: the hardware and software setups, which will be explained in the subsequent subsections.

#### Hardware setup

**Figure 7:**
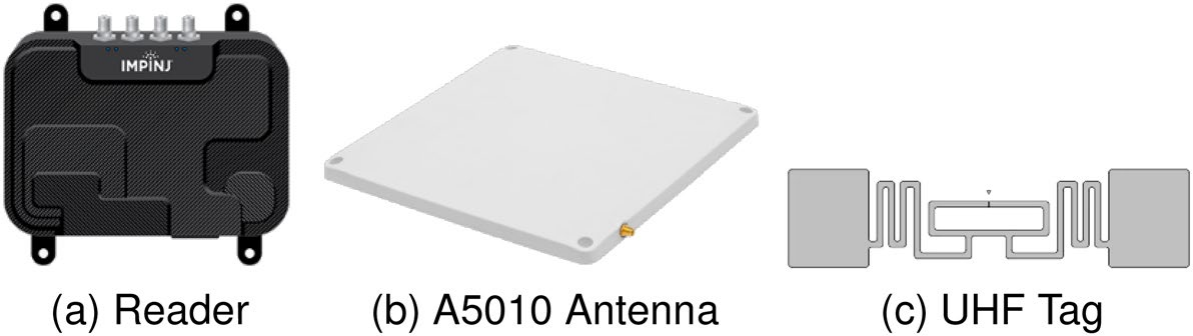
Hardware used in experimental setup.

**Figure 8:**
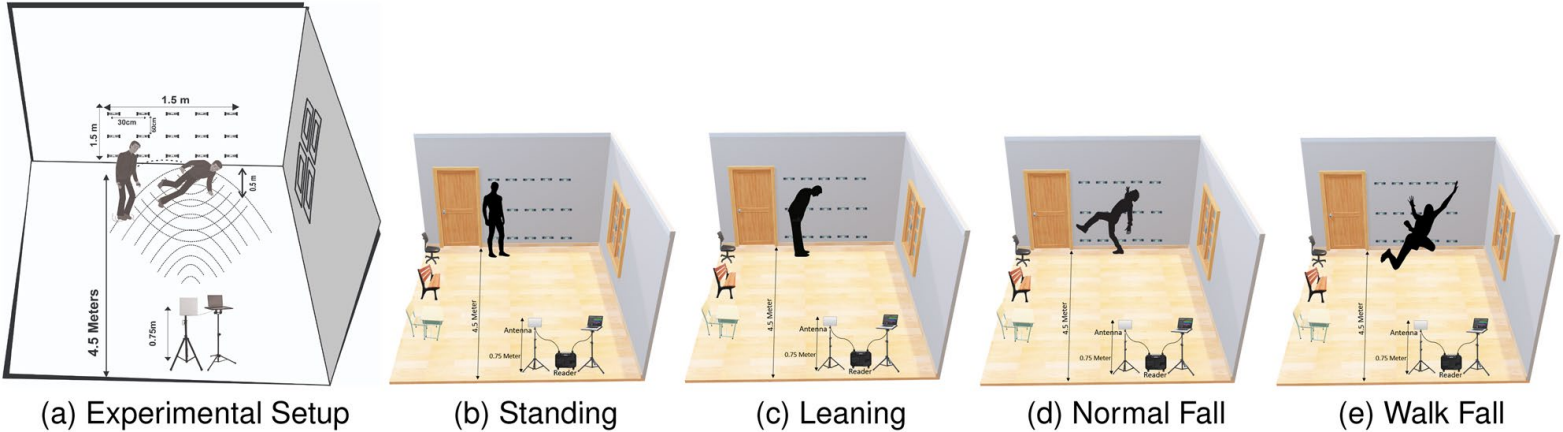
Experimental setup and fall-related activities scene.

The experimental setup utilised a laptop with the following specifications: a 64-bit Windows 10 operating system, powered by an Intel^®^ Core i7-10850H dual-core CPU operating at 2.7 GHz, and equipped with 16 GB of RAM. COTS UHF Gen-2 RFID devices were employed without any modifications. Specifically, an Impinj *R*700 reader was connected to a circularly polarized antenna measuring (250 $\times$ 250 $\times$ 14) mm, possessing an 8.0 dBi gain. This setup utilised Impinj zebra (EPC Class 1 and Gen 2) RFID tags for data collection. To maintain consistency, the RFID tags were spaced 30 cm apart and numbered from 1 to 15. They were arranged in a left-to-right and top-to-bottom fashion, as illustrated in Fig. 8a. The reader operated within the frequency range of 865–868 MHz, utilizing time-division multiplexing mode and the RF transmitter power was set at 30 dBm. The wavelength ($\lambda$) was defined at 0.34 m. Further details regarding the hardware specifications are outlined in Fig. 7.

#### Software setup

In this study, we processed the collected data and trained our fall detection transformer model using the TensorFlow 2.0 development platform and the Python programming language on a laptop. To facilitate this process, we utilised the Impinj ItemTest Software version 2.8.0 (available at https://support.impinj.com) to continuously transmit the collected measurements of RSSI and phase information from the tag array through the laptop’s RS232 serial port by the transmitter. Figure 8b–d provide a visual representation of the experimental scene, offering a contextual view of the testing environment.

### Data collection and preprocessing

In this section, we discuss the methodology applied for data collection, which involved two distinct test scenarios:

***Test Scenario 1:*** The subject performed activities with the reader and antenna positioned 3.5 m away from the subject, while the subject-*TRG-Wall* distance was maintained at 0.5 m.

***Test Scenario 2:*** The subject performed activities with the reader and antenna placed 4.5 m away from the subject, and the subject-*TRG-Wall* distance was also kept at 0.5 m.

These two carefully selected test scenarios captured different configurations and distances between the subject, reader, antenna, and the *TRG-Wall*, aiming to collect data that represented real-world situations and variations in the experimental setup.

#### Data collection

In this study, we conducted an experiment involving three subjects who varied in terms of age, weight, and height. To improve the transferability of *TFree-FD* to novel settings without manual reconfiguration, we plan to develop more generalised models. This involves training the system in multiple environments and conducting extensive testing to ensure robustness across various environments. However, due to the absence of a publicly available RFID-based dataset, we proactively created our own dataset. To ensure the reliability of our results, each subject performed five distinct activities: no-activity, standing, leaning, normal fall, and walking fall. These activities were performed at a natural pace within the designated area between the antenna and the *TRG-Wall*, as illustrated in Fig. 8. During data collection, we took meticulous care to ensure subjects consistently maintained their proximity to both the *TRG-Wall* and the antenna. To maintain experimental control and focus on individual recognition, only one subject performed the activities at a time, resulting in data from a total of 15 RFID tags. All subjects in the experiment provided their consent by signing an ethical approval form sanctioned by the institutional review board of the university of Glasgow. The data collection process resulted in a total of 2600 valid training and testing samples across two distinct scenarios. Each RFID tag was read approximately 32–36 times within a 3-s interval. Subsequently, we utilised a Python script to parse the 50 collected samples of each activity, extracting pertinent information for further pre-processing. The current study addressed five distinct daily human activities, for which phase and RSSI were two of the multimodal data. Although the dataset had sufficient data to enable the first testing, we realised that by increasing the dataset through more data collection and the use of data augmentation techniques, the model’s ability for generalisation can be improved further. The processed dataset was then employed to train and test DL algorithms. A summary of the collected dataset, including its composition and structure, is provided in Table 8.

**Table 8:** Dataset summary using *TRG-Wall*: scenarios, subjects, and activities performed.

Activity	Senario-1 (3.5 m)		Senario-2 (4.5 m)	
	RSSI	Phase	RSSI	Phase
Empty room	50	50	50	50
Standing	50	50	50	50
Leaning	50	50	50	50
Normal fall	50	50	50	50
Walk fall	50	50	50	50

#### Data preprocessing

To ensure accurate fall detection, the reflected signal from the RFID reader undergoes several essential preprocessing steps. This involves raw RSSI and phase values, carefully calibrated to address environmental and system-specific factors. The filtering methods including adaptive filtering and wavelet denoising are employed to eliminate initial phase and RSSI noise. A signal segmentation process, utilising dynamic time warping to isolate fall-relevant segments. Subsequently, comprehensive normalization standardizes the data, reducing potential biases. These steps form a robust foundation for reliable fall detection algorithms, significantly enhancing accuracy and efficiency. This preprocessing ensures subsequent stages of the fall detection algorithm operate on a refined and optimized dataset, resulting in a more dependable system.

*1. Phase Normalization:* We employ neighboring phase averaging to mitigate hardware-induced thermal noise, aligning and refining phase values for improved accuracy (Eq. 2).

2 $$\begin{aligned} \alpha = \left( \frac{4 \pi d}{\lambda } + k \cdot 2 \pi \right) \bmod (2 \pi ). \end{aligned}$$

- Noise Reduction: Gaussian smoothing complements phase normalization, reducing high-frequency noise for enhanced clarity in phase-distance relationships.
- Temporal Segmentation: Data is partitioned into discrete time intervals to isolate and analyse specific activities, including falls.
- Quality Control: Checks identify and rectifies anomalies in RFID data, ensuring data integrity through validation for missing or erroneous readings.
- Signal Alignment: Dynamic alignment synchronizes data from multiple RFID tags for temporal consistency, critical for accurate fall detection.

For data consistency, we assume $\Delta d \le \lambda = 4$ as the tag’s distance difference between consecutive sampling points, given the short time delay between rounds.

3 $$\begin{aligned} \begin{aligned} \alpha = \left( \frac{4 \pi d}{\lambda } + k \cdot 2 \pi + \Delta \alpha \right) \bmod (2 \pi ) \\ \Delta \alpha = \left( \frac{4 \pi \Delta d}{\lambda } - \Delta \phi \right) \bmod (2 \pi ). \end{aligned} \end{aligned}$$

**2. RSSI Signal Noise Reduction: **

Wavelet filtering comprises three main steps: decomposition, thresholding, and reconstruction, aimed at analyzing patterns in RSSI data. To achieve this, we utilised the discrete wavelet transform (DWT) to decompose the signal into wavelet coefficients, which capture frequency content at different scales. The DWT convolves the signal with wavelet basis functions, denoted by $\psi _{j, k}$ and $\phi _{j, k}$, operating at various scales and positions. Specifically, we employed the Coiflet-5 (coif5) wavelet to divide the raw data into five layers during the decomposition process. The decomposition equation is provided as follows:

4 $$\begin{aligned} \begin{aligned} X = \sum _{j=0}^{J-1} \sum _{k} \langle X, \psi _{j, k}^{coif5} \rangle \psi _{j, k}^{coif5} + \sum _{k} \langle X, \phi _{0, k}^{coif5} \rangle \phi _{0, k}^{coif5} \\ \end{aligned} \end{aligned}$$

During the DWT procedure, the initial signal *X* is subjected to decomposition, generating wavelet and scaling components across various levels, limited by a maximum level *J*. The inner products $\langle X, \psi _{j, k} \rangle$ and $\langle X, \phi _{j, k} \rangle$ represent correlations between the signal and the wavelet and scaling functions, respectively. This decomposition facilitates the examination of RSSI data across diverse scales and a thresholding phase contrasts each data point in the RSSI signal with a predefined threshold of $-70$ dBm. Crossing the threshold indicates noteworthy activity detection, whereas points below the threshold imply the absence of detection.

$$\begin{aligned} f(X(t)) = {\left\{ \begin{array}{ll} X(t) &{} \text {if } X(t) \ge T \\ \text {Not detect} &{} \text {if } X(t) < T \\ \end{array}\right. } \end{aligned}$$

#### Assessing the feasibility for fall detection

**Figure 9:**
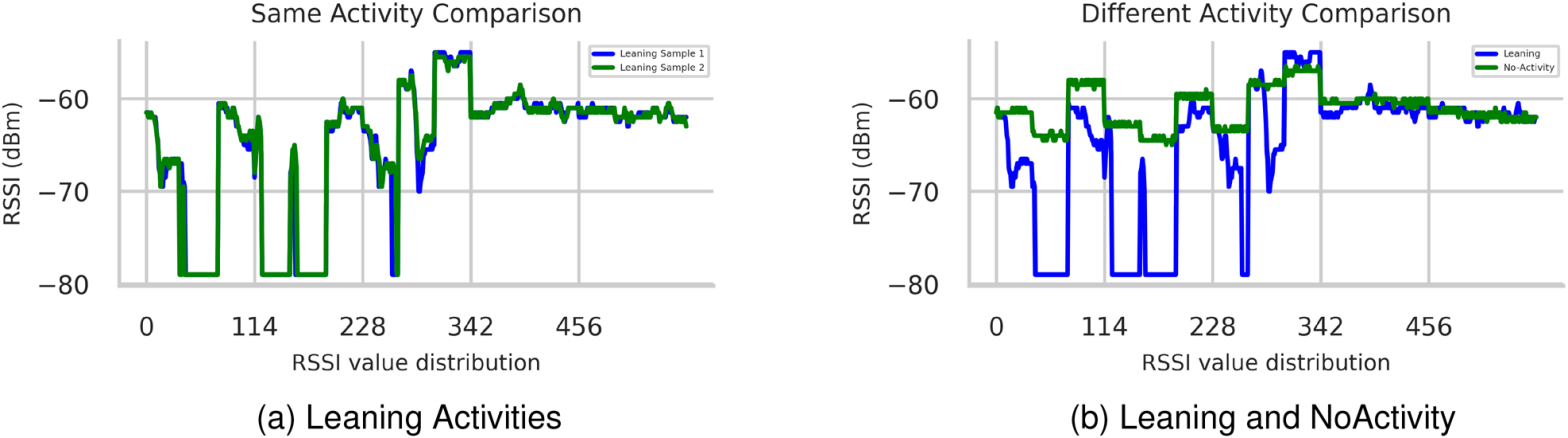
Data curves: distinguish between the same and different activities.

A feasibility study used RFID UHF tag array to detect various fall postures and prepare data for DL models as shown in Fig. 9. The study focused on how different body movements affect RFID signal waveforms, particularly RSSI. The graphical representation in Fig. 9a showed strong correlations in RSSI waveforms for repeated instances of the same leaning activity. Figure 9b demonstrated how various daily activities influence RSSI waveforms, distinguishing between leaning and no-activity. Clear fluctuations due to human activities were visible, while consistent patterns were evident without interference before or after activity. These results highlight the potential of using RFID phase and RSSI waveforms to classify human activity attributes for detection.

The feasibility analysis confirmed that RFID signals can effectively capture and differentiate various fall and fall-related activities. However, to optimize system performance, addressing signal noise and implementing precise action segmentation methods during data preprocessing are critical challenges.

## System methodology

**Figure 10:**
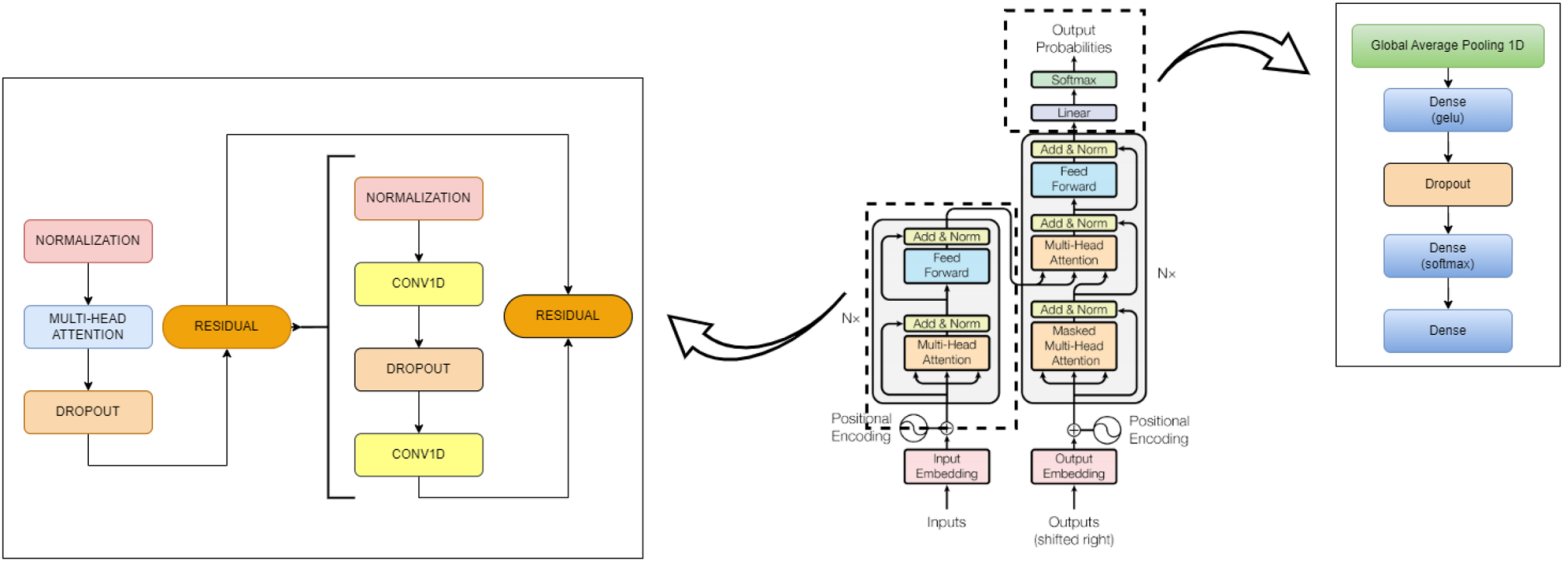
Proposed (encoder only) vs original (encoder-decoder) architectures.

**Figure 11:**
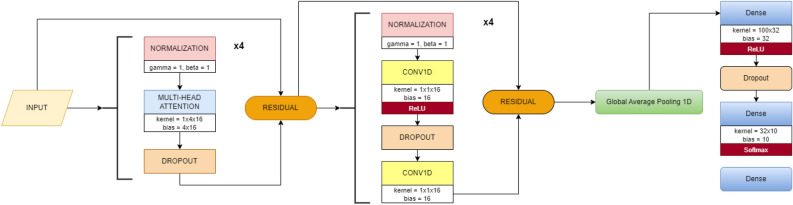
Proposed transformer encoder structure.

In this section, we employed the attention-based transformer model, which entirely avoids the use of decoder, recurrence, and convolutions. The proposed transformer architecture follows an encoder structure, effectively capturing their behavioral characteristics. To elaborate, the encoder, situated on the left side of the transformer architecture, maps an input sequence into continuous representations. Figure 11 illustrates the components of the transformer, showcasing stacked self-attention and point-wise fully connected layers in the encoder section, as depicted in the left half of Fig. 10.

### Model architecture

Below is a detailed description of the model architecture:

- Input Layer: The feature matrix (X) is fed into the model with a shape of (*number_of_features*, 1), where *number_of_features* represent the number of columns in the feature matrix.
- Transformer Blocks: The core of the model consists of four identical transformer blocks, each comprising the following components:
  LayerNormalization followed by a *MultiHeadAttention* layer and a *Dropout* layer.
  The output of the Dropout layer is combined with the input of the transformer block using a residual connection.
  Another LayerNormalization layer follows.
  Two Conv1D layers, where the first one employs *ReLU* activation. The output of the second Conv1D layer is merged with the output of the first LayerNormalization layer using another residual connection.
- GlobalAveragePooling1D Layer: This layer is implemented to reduce the model’s output dimensions.
- Dense Layers: The standard fully connected layers with *gelu* activation function are used. The number and sizes of these layers are determined by the *mlp_units* parameter.
- Output Layer: The final layer of the model is another Dense layer, containing the same number of neurons as the number of classes. The activation function used is *softmax*, making this model suitable for multiclass classification tasks.

### Transformer architecture encoder structure

The encoder consists of $N=4$ identical layers, each containing two sublayers:

- The first sublayer uses a 4-headed multi-head self-attention mechanism, with each head processing unique linear projections of queries, keys, and values, contributing to the final output.
- The second sublayer includes two *Conv1D* layers, with the first layer utilizing a *ReLU* activation function.

5 $$\begin{aligned} \textrm{FFN}(\textrm{x})={\text {ReLU}}\left( \textrm{W}_1 \textrm{x}+\textrm{b}_1\right) \textrm{W}_2+\textrm{b}_2 \end{aligned}$$

In the transformer architecture, each sublayer is augmented with a residual connection and followed by a *LayerNormalization* layer. This ensures proper normalization of the sublayer’s input, denoted as ‘x’, and its output, *sublayer(x)*. However, the transformer architecture lacks inherent positional awareness due to its non-recurrent nature. To address this, positional encodings are introduced by adding them to the input embeddings, providing essential positional information.

6 $$\begin{aligned} \text{ LayerNormalization } (\textrm{input}+{\text {sublayer}}(\textrm{output})) \end{aligned}$$

### The transformer multi-head attention

The attention function operates on query and key-value pairs, generating an output represented as vectors. The output is obtained through a weighted summation of values, with the weights determined by a compatibility function applied to the query and key. In order to retain the spatial information of RSSI and phase data, we avoid the use of positional embedding^64^.

The feature extraction module of the transformer comprises two sub-layers: multi-head self-attention and multiscale residual CNN with adaptive scale attention. These sub-layers utilise a residual connection (Add)^65^ and layer normalization (LayerNorm)^66^. Self-attention effectively handles long-term dependencies in sequences, surpassing the limitations of RNN and LSTM models. It captures global information from the entire sequence and overcomes the constraints of CNN’s perception field and reliance on time-domain information. In the transformer, the input contains (*queries, keys, and values*), each having dimensions $d_k$ and $d_v$ respectively. These inputs undergo a *softmax* function to derive attention weights, which are then used to scale the values through weighted multiplication. The *multi-head* attention blocks in the transformer execute a scaled dot-product attention operation. This process can be summarized as follows:

7 $$\begin{aligned} {\text {Attention}}(\textrm{Q}, \textrm{K}, \textrm{V})={\text {softmax}}\left( \frac{\textrm{Q} \textrm{K}^{\textrm{T}}}{\sqrt{\textrm{d}_{\textrm{k}}}}\right) \textrm{V} \end{aligned}$$

The attention mechanism in the transformer enables capturing dependencies between data sequence elements and extracting pertinent features for subsequent processing.

8 $$\begin{aligned} \begin{aligned} {\text {MultiHead}}(Q, K, V) = {\text {Concat}}({\text {head}}_1, \ldots , {\text {head}}_{\textrm{h}}) W^O \\ \text {where } {\text {head}}_{\textrm{i}} = {\text {Attention}}(Q W_i^Q, K W_i^K, V W_i^V) \end{aligned} \end{aligned}$$

In this study, we employ 8 parallel attention layers (or heads) with $h = 8$, each having 64 dimensions. This reduction in dimensionality ensures a computational cost similar to that of single-head attention with full dimensionality.

### Architecture comparison

The proposed architecture is adapted from Vaswani et al.^29^ transformer model, as depicted in Fig. 11. However, it differs from the comprehensive transformer model shown in Fig. 10 as follows:

1. Encoder Only vs. Encoder-Decoder: The proposed model exclusively uses transformer *Encoder* blocks, similar to BERT^64^. In contrast, the original transformer incorporates both *Encoder* and *Decoder* blocks, primarily for sequence-to-sequence tasks like translation.
2. Global Average Pooling: The proposed architecture includes a *GlobalAveragePooling1D* layer after the transformer blocks to reduce output dimensionality for classification tasks.
3. Fully Connected Layers: The proposed architecture introduces *Dense* layers with dropouts after the transformer blocks, setting it apart from the original transformer.
4. Output Layer: For multi-class classification, the proposed architecture utilises a Dense layer with *softmax* activation, while the original transformer uses a *final linear* layer followed by softmax for sequence-to-sequence tasks, predicting the next word in a sequence.
5. Positional Encoding: Unlike the original transformer model, the proposed architecture does not employ positional encoding.

### Ablation studies

The ablation study systematically analyses components affecting system performance. It assesses user diversity and location impacts, explores antenna height effects on RFID tag reading, and examines the advantages of combining RSSI and phase data. These insights contribute to robust fall detection.

#### Impact of user diversity and location on performance

**Figure 12:**
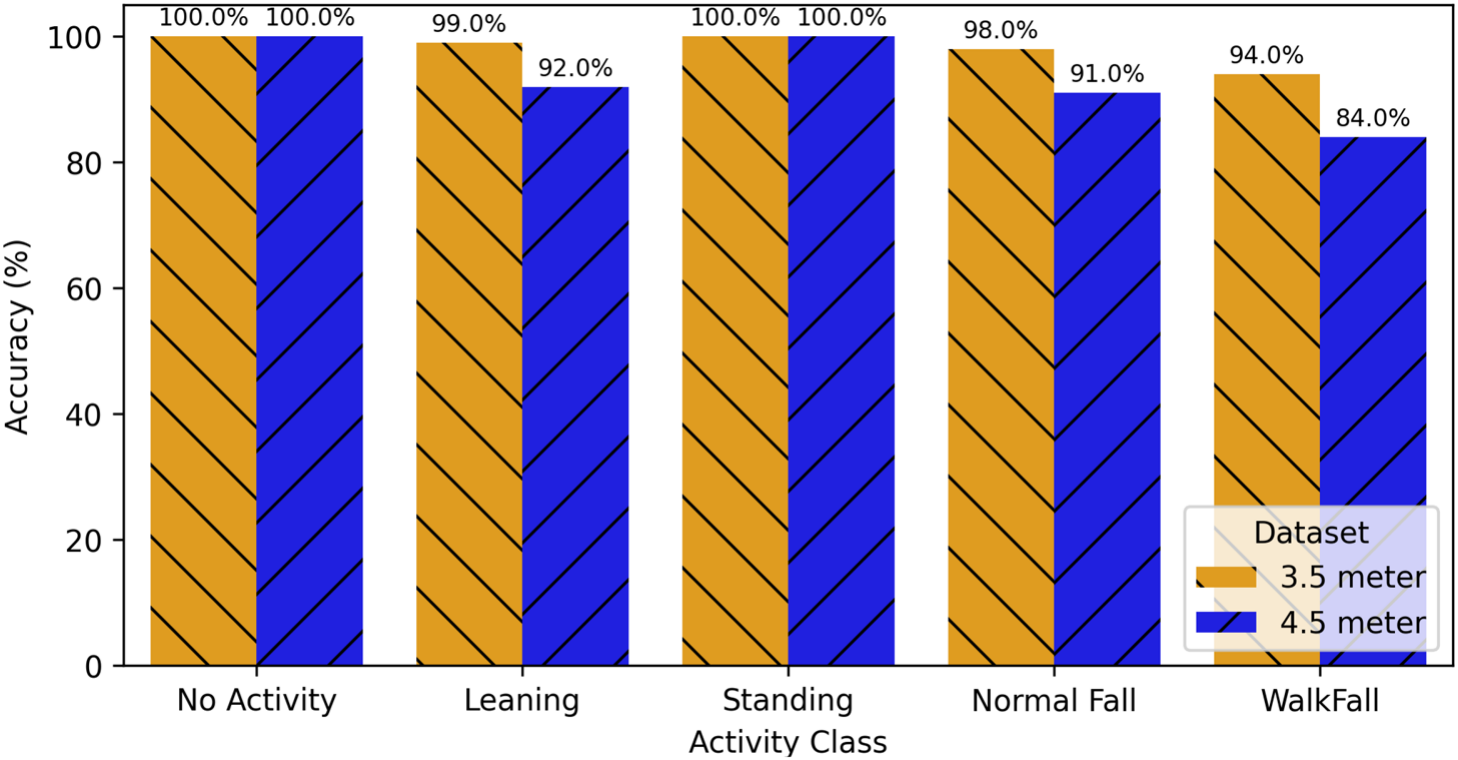
Comparison of class-wise accuracies between 3.5 and 4.5 m datasets.

To assess the system’s stability and generalization ability, we conducted experiments with multiple subjects, utilising distinct fall-related activity data without any training or validation sets. Under the same system deployment mode, there are five distinct activities, including falls, with predefined distances of 3.5 and 4.5 m between the antenna and the tag. Figure 12 illustrates the model’s adaptability for fall detection across different activities. We further examined the impact of the target subject’s position within the fall perception system on detection accuracy. At 3.5 m, accuracy exceeded $98\%$, with the exception of WalkFall achieving $94\%$. This variation indicates that RFID signals are influenced by the multipath effect in the physical environment, leading to a slight decline in recognition accuracy for non-preset positions. Nevertheless, the system maintains satisfactory recognition performance. This underscores the robustness of the proposed fall detection method, ensuring optimal performance in indoor environments.

#### Impact of antenna height

This study explores the impact of antenna height on RFID tag reading performance. Antenna placement has a significant effect on range and precision. Two placements were tested: The initial setup mounted the antenna on a ground-level wall measuring 1.5 $\times$ 1.5 m$^2$. This affected the reading of top-row tag RSSI/phase data. Adjusting the height to 0.75 m, aligning with the wall’s center and LoS, aimed to extend the range with minimal accuracy impact. However, raising it further to 1.5 m weakened the signal to the lower tag row due to insufficient reader strength. Results suggest maintaining the default 0.75 m height for optimal system performance.

#### Impact of multimodal analysis

**Figure 13:**
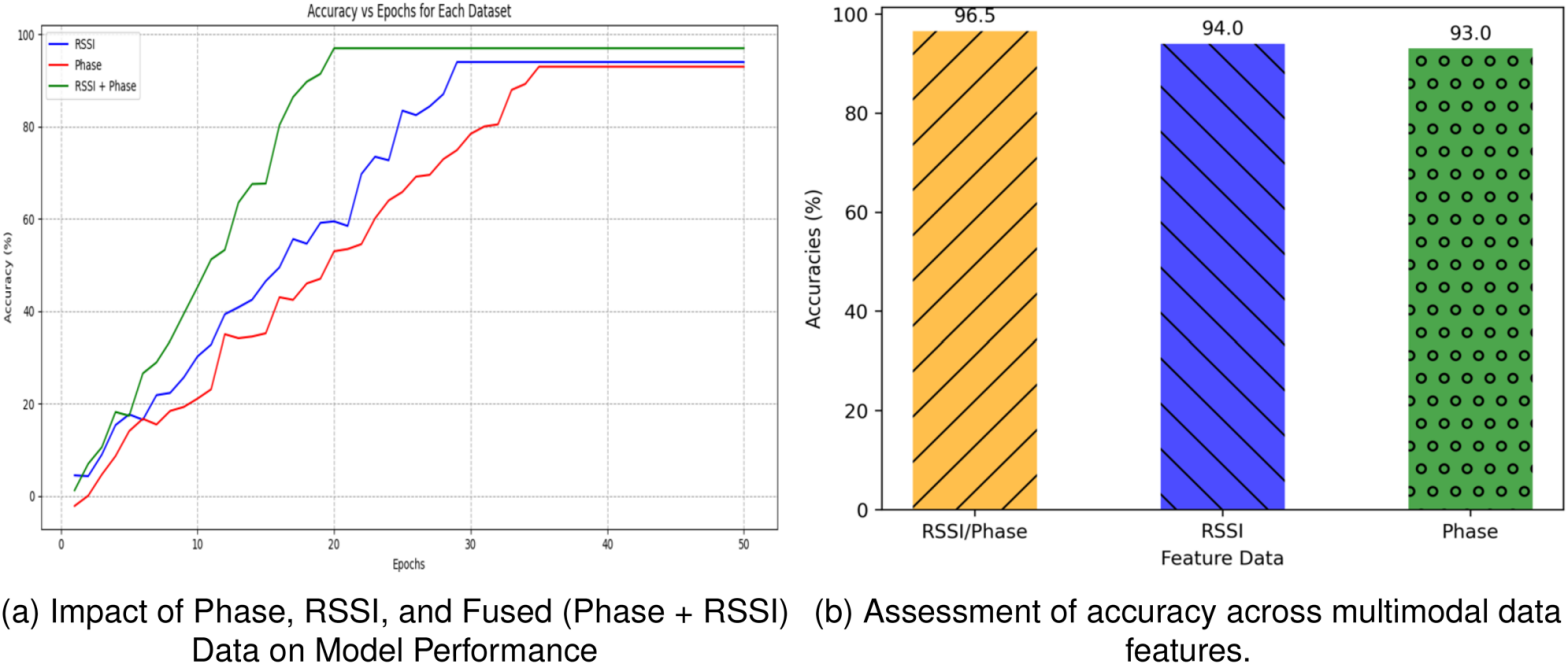
Comparison of model performance based on different data representations.

In this section, we explore the impact of multimodal features on recognition accuracy. To evaluate the system’s performance, we initially select RSSI, phase, and fusion signals as sample data. The incorporation of multiple signal types aims to harness a broader spectrum of features, potentially leading to enhanced recognition accuracy. We proceed by training and validating the model using three datasets. The accuracy curve of the test set during the training process is depicted in Fig. 13a. The results clearly indicate that, under identical training conditions, the combination of RSSI and phase data as feature inputs facilitates faster convergence and achieved higher accuracy during the training process. Our experimental findings demonstrate that the fused feature data outperforms the individual phase or RSSI features in terms of fitting speed and final accuracy, as illustrated in Fig. 13b. This superiority arises from the fact that the phase signal is more sensitive to environmental factors, while the RSSI resolution diminishes with increasing distance from the antenna. Therefore, employing fused data features becomes preferable for the fall detection system.

Figure 13b demonstrates that combining RSSI and phase data yields higher accuracy compared to using either RSSI or phase data alone. Building on this finding, we investigate the impact of distance and the number of tags on the system’s recognition performance. The accuracy results are presented in Table 3. The experiment reveals that increasing the distance from 3.5 to 4.5 adversely affects the model’s recognition effectiveness. As the fall detection system prioritizes swift recognition, incorporating additional RFID tags in close proximity to the activity area significantly reduces the system’s sampling rate, while also imposing a higher computational burden on the model. Therefore, to maintain a reasonable computational cost and meet the fall detection requirements in indoor scenarios, we have adopted a layout with fifteen tags, as confirmed in this study. This configuration satisfies the system’s needs and ensures efficient fall detection.

## Conclusion, limitations and future directions

This paper investigates the impact of RFID tag signals on monitoring the activity of the elderly in a contactless manner. The study focuses on using phase and RSSI features as inputs for a transformer-based deep neural network to develop a system for detecting falls. Future work aims to integrate online learning to enable the system adaptation to dynamic environments. This will improve the robustness and flexibility of *TFree-FD*, allowing it to maintain high accuracy even as environmental conditions change. We collected a dataset with multimodal data relating to five distinct daily human activities, including phase and RSSI. The preprocessing steps of noise reduction and normalisation were applied to the data before to the transformer model training. In two distinct scenarios, the trained model achieved a high accuracy of $96.5\%$. The experimental results demonstrate the robustness of the contactless fall detection system against variations in users and locations. However, the proposed fall detection method in this paper still has certain limitations that require further improvement in future research.

## Future work

This paper investigates the impact of RFID tag signals on monitoring the activity of the elderly in a contactless manner, focusing on using phase and RSSI features as inputs for a transformer-based deep neural network to develop a system for detecting falls. Future work aims to integrate online learning to enable the system adaptation to dynamic environments, improving the robustness and flexibility of *TFree-FD*, and maintaining high accuracy even as environmental conditions change. Additionally, future research will extend the current study to include the recognition of pre-fall behaviours such as staggering and dizziness. By incorporating these features, we aim to increase the system’s predictive capabilities and provide early warnings to prevent falls. Extensive testing in multi-environments, including residential homes, care facilities and public spaces, will also be conducted to ensure the model’s robustness and reliability across different settings, thereby enhacing its practical applicability. To further improve the model’s generalisation capability, future studies will focus on augmenting the dataset size by collecting additional data from diverse locations and subjects. We will explore various data augmentation techniques, such as generating synthetic data, to intentionally increase the size and variety of the dataset. Overall, future improvements will focus on certain key areas: integrating online learning for real-time adaptation, conducting extensive multi-environment testing, and reducing dependence on labelled data through semi-supervised or unsupervised learning techniques. These directions are essential for enhancing the system’s practical applicability and ensuring its reliability in real-world scenarios.

We collected a dataset with multimodal data relating to five distinct daily human activities, including phase and RSSI. The preprocessing steps of noise reduction and normalisation were applied to the data before to the transformer model training. In two distinct scenarios, the trained model achieved a high accuracy of $96.5\%$. The experimental results demonstrate the robustness of the contactless fall detection system against variations in users and locations. However, the proposed fall detection method in this paper still has certain limitations that require further improvement in future research.

1. **Multi-subject Fall Detection:** The proposed method detects falls in one person only and doesn’t address detecting falls in multiple individuals due to challenges with contact-less deployment and signal separation. Research is ongoing to explore contactless real-time fall detection for multiple users using human body feature signals.
2. **Collecting Authentic Human Fall Data:** The collected fall data may not fully represent real falls due to challenges in collecting authentic human fall data. Falls can be categorized as object-related or fainting-induced, with real falls being sudden and unpredictable. Controlled environments in experiments capture diverse fall actions but differ from real falls. Training the system on simulated data may lead to deviations when detecting sudden falls in real bodies. Collecting more real fall data is vital for enhancing the system’s accuracy in detecting unforeseen falls.
3. **Scalability and Application Potential: ** The proposed system, initially tested in a $10 \times 10$ $m^2$, excels at detecting falling-related activities within designated area. The contactless RFID-based scheme, known for its remarkable scalability, can easily extend its coverage to larger spaces by adding more tags and antennas. This scalability not only maintains a low average cost but also positions it as an ideal solution for healthcare facilities, nursing homes, smart homes, and other environments where cost-effectiveness and non-intrusiveness are essential, making RFID tags a viable and adaptable solution for widespread adoption.
4. **Environment Dependency and Training Costs:** The phase and RSSI signals depend on the deployment environment of the system, which can vary in practical applications (e.g., hospitals, homes, and nursing homes). Different objects and environments can reduce accuracy. Adding sample data or training models in new environments incurs high learning and training costs for RFID. Future work should prioritize passive sensing across diverse environments and devices.

## Conclusions

This study presents an innovative RFID-based method for contactless fall detection in the elderly, utilising strategically placed passive UHF RFID tag array in two distinct scenarios. The setup mimics real-world conditions by positioning the tag array half meter from the subject, ensuring realistic detection and pseudo localisation based on tag blockage and the activity performed in front of the tags. Unlike conventional approaches that require cumbersome wearables, our system operates seamlessly through tag querying and minimal data processing. By employing a transformer model and early fusion technique, we achieved an impressive average accuracy rate exceeding $96.5\%$, demonstrating the superior efficacy and practicality of our approach over conventional methods such as CNN, RNN, and LSTM. This highlights the potential of our method to revolutionise fall detection, providing a highly effective, contactless solution that enhances the safety and well-being of elderly individuals.

## Data Availability

The datasets utilised and analysed in the present study can be obtained from the corresponding author upon reasonable request.
